# Systematic comparative analysis of strand-specific RNA-seq library preparation methods for low input samples

**DOI:** 10.1038/s41598-021-04583-z

**Published:** 2022-02-02

**Authors:** Swati Naphade, Rajat Bhatnagar, Victor Hanson-Smith, Irene Choi, Alice Zhang

**Affiliations:** 1 Verge Genomics, South San Francisco, CA USA; 2 Present Address: Fountain Therapeutics, South San Francisco, CA USA

**Keywords:** Next-generation sequencing, RNA sequencing

## Abstract

Despite the recent precipitous decline in the cost of genome sequencing, library preparation for RNA-seq is still laborious and expensive for applications such as high throughput screening. Limited availability of RNA generated by some experimental workflows poses an additional challenge and increases the cost of RNA library preparation. In a search for low cost, automation-compatible RNA library preparation kits that maintain strand specificity and are amenable to low input RNA quantities, we systematically tested two recent commercial technologies—Swift RNA and Swift Rapid RNA, presently offered by Integrated DNA Technologies (IDT) —alongside the Illumina TruSeq stranded mRNA, the de facto standard workflow for bulk transcriptomics. We used the Universal Human Reference RNA (UHRR) (composed of equal quantities of total RNA from 10 human cancer cell lines) to benchmark gene expression in these kits, at input quantities ranging between 10 to 500 ng. We found normalized read counts between all treatment groups to be in high agreement. Compared to the Illumina TruSeq stranded mRNA kit, both Swift RNA library kits offer shorter workflow times enabled by their patented Adaptase technology. We also found the Swift RNA kit to produce the fewest number of differentially expressed genes and pathways directly attributable to input mRNA amount.

## Introduction

RNA sequencing (RNA-seq) is a common technology to profile the transcriptome of cells and observe gene expression variation in both natural and perturbed conditions. Typical experiments require the isolation of RNA from samples under study and the preparation of cDNA libraries, followed by sequencing and bioinformatic analysis. The choice of the RNA-seq library preparation method is dictated by several factors, such as cost, RNA quality, and RNA input amount. Low cost methods suitable for low input RNA amounts are of particular interest, as cost and material are two key limiting factors in high throughput gene expression experiments. Several studies have focused on bulk RNA-seq library preparation methods for low RNA inputs^1–4^. Some of these methods were found to perform relatively poorly across several metrics when compared to the Illumina TruSeq kit de facto standard, while others were typically expensive or incompatible with automation, and thus not ideal for high throughput experiments designed for gene target validation or compound screening efforts.

Here we compare three different bulk RNA-seq library preparation methods, aiming to understand differences in both absolute and differential gene expression. We selected two recently developed methods, the Swift and the Swift Rapid library preparation methods, as well as the de facto standard, the Illumina TruSeq method, as a reference for comparison (Table 1). The Swift and the Swift Rapid kits, currently offered by Integrated DNA Technologies (IDT), are designed for inputs as low as 10 ng and 50 ng total RNA, respectively, while 100 ng total RNA is the minimal recommended input for the Illumina TruSeq kit. Throughout this work we compare the methods using the Universal Human Reference RNA (UHRR)^2,5,6^ to ensure high coverage across the transcriptome and avoid bias towards any subset of genes. All three library preparation methods can be automated in order to increase productivity, ensure greater reproducibility, and minimize human error.

**Table 1:** Library preparation kit comparison and cost analysis.

Vendor	Kit	Method	**Cost/sample	Time (h)	Input RNA range	mRNA enrichment method	ncRNA	Stranded	Low RIN Samples	Automation compatible
Illumina	TruSeq stranded mRNA	dUTP second strand incorporation	$65	9.0	100 ng–1 μg	polyA-selection		✓		✓
Swift Biosciences	Swift RNA library kit	Adaptase methods	$40	4.5	10 ng–1 μg	polyA-selection or ribodepletion	✓	✓	✓	✓
	Swift Rapid RNA library kit	Adaptase methods	$30	3.5	100 ng–1 μg	polyA-selection or ribodepletion	✓	✓		✓

**Cost/sample includes costs of plastic consumables, the upstream poly-A selection kit, library prep kit, SPRI beads, and KAPA qPCR quantification kit.

As bulk RNA-seq library preparation methods are known to be sensitive to RNA input amount, in this study we tested the three different methods with varying input amounts of RNA. While RNA is typically abundant in in vivo experiments, many human stem cell model systems that are used to mimic disease progression in human patients produce limited amounts of RNA. Therefore, methods capable of producing high quality libraries from small amounts of RNA (< 100 ng) are especially valuable.

An additional consideration in library preparation is strand specificity. The human genome comprises several overlapping genomic loci that are transcribed from opposite strands and encode distinct genes with different functions (commonly called overlapping genes). As such, the ability to distinguish which strand is expressed in a sample is of crucial interest. A serious shortcoming of the first generation RNA-seq protocols was that they did not retain strand specificity, making it challenging to quantify gene expression levels for overlapping genes^7^. Newer generation RNA-seq methods however do retain strand information, thereby resolving read ambiguity in overlapping genes and producing more accurate and higher quality transcriptomic data^7–9^. Strand specificity is incorporated into these RNA-seq protocols by (i) ligation of 3′ pre-adenylated and 5′ adapters, (ii) labeling of the second strand with dUTP followed by enzymatic degradation, (iii) template-switch attachment of the 3′ adapter (the Peregrine method), or (iv) Breath Adapter Directional Sequencing (BrAD-seq)^7,8^. All three RNA-seq methods used in this study are strand-specific. While Illumina TruSeq library kit uses dUTP labeling to degrade the second strand, Swift and Swift Rapid RNA kits maintain strand-specificity by making only one functional strand which is immediately ligated with a 3’ truncated adapter.

A crucial step in library preparation for typical RNA-seq experiments is the enrichment of mRNA. The mammalian transcriptome comprises multiple types of coding and non-coding (nc) RNA species, including small (micro) and long ncRNA, ribosomal RNA (rRNA), and messenger RNA (mRNA). While most RNA-seq experiments entail isolation of mRNA species, small and long ncRNAs which lack protein-coding potential have emerged as important regulators of gene expression networks^10^. These RNA subtypes can be isolated and enriched from total cellular RNA using protocols such as the oligo (dT) selection method to select for polyadenylated mRNA transcripts, ribodepletion protocols to remove ribosomal RNAs, or size selection to isolate small ncRNA. Ribodepletion is an ideal method to isolate low RIN (RNA Integrity Number) mRNA from degraded low-quality biological samples. Since in our experiments, we were interested in mRNA quantification and the input UHRR was verified to be high quality with RIN greater than 9.0, oligo (dT) selection was used for all three kits. For the Illumina TruSeq library prep, mRNA was isolated from total RNA as per the protocol guidelines, whereas for the Swift and Swift Rapid RNA library prep methods, an upstream NEBNext oligo (dT) selection module was used to isolate mRNA from total RNA.

**Figure 1:**
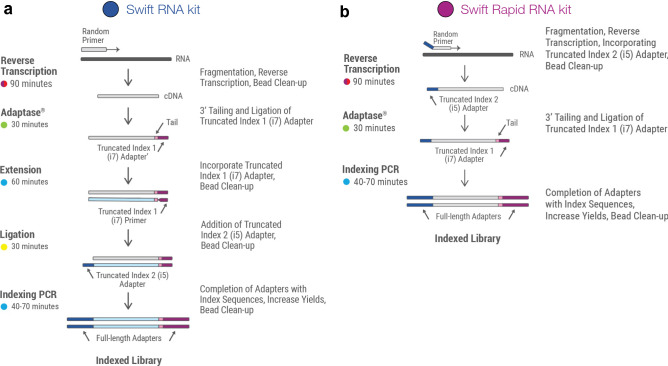
Workflows for library preparation using (**a**) the Swift RNA kit and (**b**) the Swift Rapid RNA kit.

The three library preparation methods under consideration have different workflows. The Illumina TruSeq mRNA workflow includes mRNA fragmentation and synthesis of first strand cDNA using Superscript II Reverse Transcriptase, followed by second strand cDNA synthesis. The 3′ ends of the resulting double stranded cDNA (dsDNA) inserts are then adenylated. This is followed by adapter ligation and finally enrichment of DNA fragments^11^. This workflow takes 9 h. The Swift and Swift Rapid RNA library preparation methods, on the other hand, employ proprietary Adaptase technology^12^ (US Patent No. 9,896,709) that reduces the library preparation time to 4.5 h and 3.5 h, respectively (Fig. 1). In the Swift workflow, fragmented mRNA is reverse transcribed into first strand cDNA using random hexamer primers. A truncated adapter is then directly ligated on to single stranded DNA (ssDNA), thereby eliminating the need for second strand synthesis. This is followed by extension, ligation, and finally, indexing PCR. Compared to traditional library prep methods, the Adaptase technology provides improved read mapping, reduced artifactual reads, enhanced throughput, and increased library complexity and coverage uniformity^12^. The Swift Rapid RNA library prep is an expedited version of this workflow that does not include the extension and ligation steps. A random primer conjugated to a truncated adapter is used to prime the Reverse Transcription reaction. Adaptase then adds the second 3′ truncated adapter onto the first strand ssDNA. Amplification of final libraries is performed using indexing PCR.

In our work, the final libraries prepared using the Illumina, Swift, and Swift Rapid workflows were sequenced at an average read depth of 20 million reads per library (“Methods”). These libraries were the basis of our computational analysis of the three methods.

## Results

### All three workflows produced high quality libraries

We selected three library preparation methods for comparison, the Swift RNA Library Prep, Swift Rapid RNA Library Prep, and Illumina TruSeq Stranded mRNA Library Prep. UHRR, a pool of equal quantities of RNA prepared from 10 different cancer cell lines, was used as input RNA. To ensure a fair comparison applicable to common laboratory settings, we prepared multiple libraries with input amounts of total RNA in ranges recommended by the manufacturer (Table 2). In brief, we prepared the following libraries with varying input UHRR (total RNA):

- Swift RNA kit: 10, 50, and 100 ng
- Swift Rapid RNA kit: 50, 100, and 200 ng
- Illumina TruSeq Stranded mRNA kit: 50, 100, 200, and 500 ng

**Table 2:** Overview of different RNA library preparation kits and conditions analyzed in this study.

RNA Library kit	RNA type	Input (ng of total RNA)	Library Name	PCR cycles
lllumina TruSeq Stranded mRNA kit	mRNA	50	50_ng_A##_IL	15
	mRNA	100	100_ng_A##_IL	15
	mRNA	200	200_ng_A##_IL	15
	mRNA	500	500_ng_A##_IL	15
Swift RNA library kit	mRNA	10	10_ng_U###_S	20
	mRNA	50	50_ng_U###_S	17
	mRNA	100	100_ng_U###_S	13
Swift Rapid RNA library kit	mRNA	50	50_ng_U###_SR	15
	mRNA	100	100_ng_U###_SR	14
	mRNA	200	200_ng_U###_SR	13

**Figure 2:**
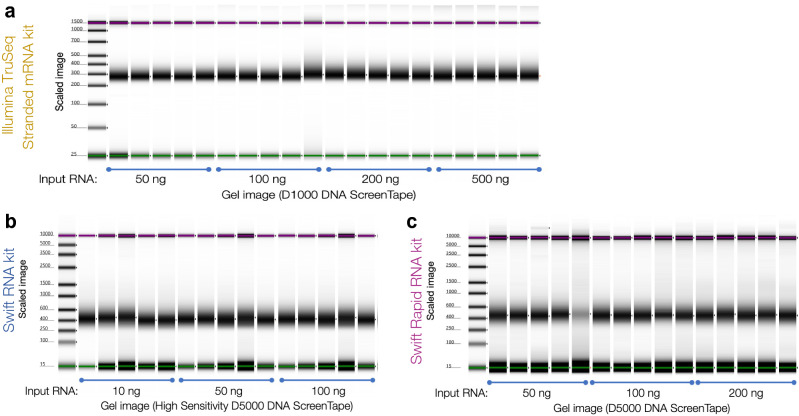
Agilent Tapestation 4200 electropherograms of all libraries prepared using the (**a**) Illumina TruSeq stranded mRNA kit, (**b**) Swift RNA kit, and (**c**) Swift Rapid RNA kit. All methods produced high-quality libraries.

Each of these 10 groups was tested in replicates of 5 samples each, for a total of 50 samples. We found all three workflows produced high quality libraries at each input amount (Fig. 2). After sequencing, 10 million reads were randomly selected from each library for analysis. This subsampling was performed to remove any effects due to the variation in sequencing depth.

### Normalized gene expression is comparable across surveyed library preparation methods

**Figure 3:**
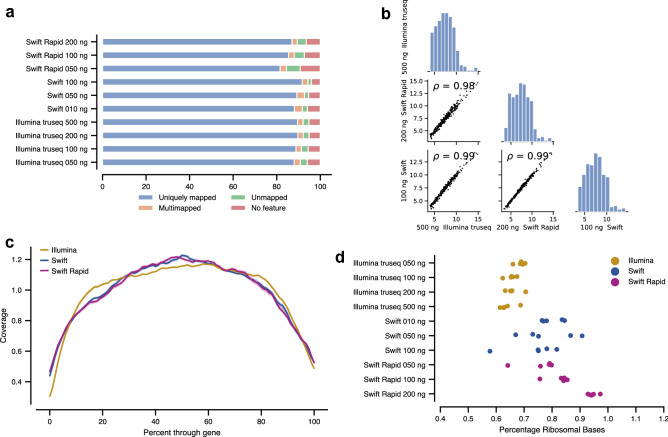
(**a**) 10 million randomly selected reads from each sample were aligned and quantified by STAR. For each library preparation method and input amount, > 80% of reads mapped uniquely to a single gene (average over samples). (**b**) Normalized read counts from identical samples prepared using three methods (Illumina TruSeq with 500 ng input, Swift RNA with 100 ng input, and Swift Rapid RNA with 200 ng input) are highly correlated. (**c**) Read coverage through genes is consistent across all methods (lines shown are averages of samples for each library preparation method). (**d**) The fraction of reads that map to ribosomal genes is less than 1% for all samples.

Quality control metrics showed that all libraries prepared were sufficient. Alignment to the human genome^13^ showed high mapping efficiency, with greater than 80% of reads uniquely mapped to genes (Fig. 3a), and expression measured for 12,000 protein coding genes. We further compared absolute gene quantifications from the three methods at the highest input amount of RNA. After normalization by the regularized log transform^12^, we found high agreement between all methods, with a Pearson correlation of > 0.97 between measured gene expression in all pairs of methods (Fig. 3b). Similar results were found for libraries prepared from samples with lower input amounts (Supplemental Fig. 1). Furthermore, using the open source software Picard Tools^14^ we observed uniform read coverage across gene base pair position, with little to no 3′ or 5′ bias (Fig. 3c). We then checked if all library preparation methods were successful at effectively depleting ribosomal RNA, finding that fewer than 1% of reads were mapped to ribosomal genes in any sample (Fig. 3d).

Additional quality control checks confirmed that the libraries produced were of similar quality. For all three preparation methods, > 90% of the reads for each library mapped to the correct strand (Supplemental Fig. 2a). We did not observe any differences in library complexity, measured by the number of genes detected above a specified threshold (Supplemental Fig. 2b). Additionally, gene expression in each library was not notably correlated with either gene length or gene GC content (Supplemental Fig. 2c,d).

To better understand how well these methods agreed with each other, we determined the set of 250 highest and the set of 250 lowest expressed genes for each library using the normalized counts. The set of 250 highest expressed genes was similar for all methods, with a Jaccard similarity coefficient greater than 0.69 for all pairwise comparisons (Supplemental Fig. 3a). The set of 250 lowest expressed genes exhibited more variability. Within each method (Illumina TruSeq, Swift RNA, and Swift Rapid RNA), we observed similarly high levels of agreement among the input RNA amounts, but across treatment groups, there were widespread differences with pairwise Jaccard similarity ranging between 0.33 and 0.61 (Supplemental Fig. 3b).

### Normalized expression of reference genes is robustly recovered with different methods

We then examined the expression of human housekeeping reference genes as an additional quality measure. Human housekeeping genes are important for several fundamental cellular processes, and therefore are expected to maintain constant expression levels across all cells and under all conditions. However this is not always true. Sources of bias in housekeeping gene detection can be attributed to several reasons: (i) genes having several splice variants could have different expression levels, (ii) duplicative regions (including pseudogenes) may complicate read alignments, and (iii) lower expression of upstream exons due to imperfect reverse transcription resulting in partial cDNA molecules^15^. Using a set of criteria to define human housekeeping genes, Eisenberg and Levanon identified 3,804 such housekeeping genes, and further proposed a short list of eleven highly uniform and strongly expressed housekeeping genes that may be used for calibration in laboratory settings.

**Figure 4:**
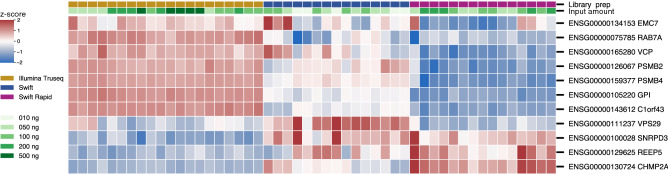
Standardized expression using regularized counts for housekeeping genes (rows) in each sample (columns). Red or blue boxes in the heatmap indicate that a gene's expression in a sample is above or below the mean over all samples, by the indicated number of standard deviations, respectively. Rows and columns are hierarchically clustered using the Euclidean metric. The differences in gene expression are mostly within a single standard deviation from the mean.

We looked at the normalized expression of these housekeeping genes and found good agreement between the three kits and also across different input amounts from the same kits with few minor variations (Supplemental Fig. 4). Figure 4 shows standardized expression (mean zero, standard deviation 1 for each gene) of the regularized counts. Briefly, a red (respectively blue) box in the heatmap indicates that a gene's expression in a sample is above (respectively below) the mean gene expression over all samples, by the specified number of standard deviations. These results show that while there are consistent differences in gene expression due to the method of library preparation, these differences are small. With few exceptions, the differences in gene expression are within a single standard deviation from the mean.

**Figure 5:**
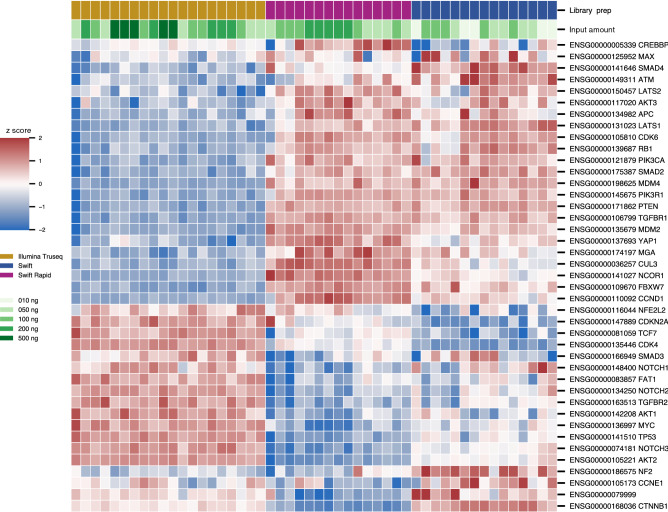
Standardized expression using regularized counts for oncogenic genes (rows) in each sample (columns). Red or blue boxes in the heatmap indicate that a gene's expression in a sample is above or below the mean over all samples, by the indicated number of standard deviations, respectively. Rows and columns are hierarchically clustered using the Euclidean metric. The differences in gene expression are mostly within a single standard deviation from the mean.

UHRR is composed of RNA from ten different cancer cell lines. Therefore, we checked the expression of typical genes in oncogenic signaling pathways. An integrated analysis of genetic alterations in > 9,000 tumors from 33 cancer types profiled by The Cancer Genome Atlas (TCGA) yielded 10 curated signaling pathways^16^. Altogether the 42 most commonly altered oncogenic genes were identified from these signaling pathways. We looked at the expression of these 42 reference genes across the three library prep methods, finding the normalized expression to be constant across the three methods and varying input mRNA amounts (Supplemental Fig. 5). The heatmap of standardized expression of these genes (Fig. 5) again showed that with a few minor exceptions, the differences in gene expression are small and within a single standard deviation from the mean.

### Differential expression of genes and pathways is sensitive to changes in mRNA input amount

From these analyses, we concluded that absolute gene expression, after appropriate normalization, was comparable across the library kits and input mRNA amounts. We then turned to the more precise task of quantifying the number of genes whose expression varied due to technical considerations. As many high throughput experiments are limited by amounts of mRNA, we focused on how gene expression changes with respect to the input amount of mRNA for each library preparation method. For this, we used a standard statistical test of differential gene expression^20^ (“Methods”).

**Figure 6:**
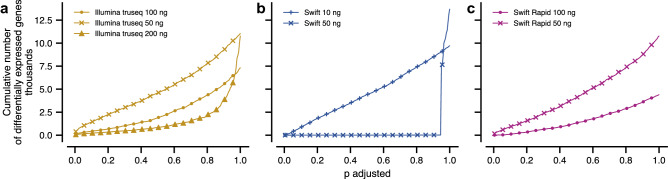
Comparison of differential gene expression results. Number of differentially expressed genes at a given level of significance depends on input amount. Differential expression computed with respect to reference samples prepared with (**a**) the Illumina TruSeq kit with 500 ng input, (**b**) the Swift RNA kit with 100 ng input, and (**c**) the Swift Rapid RNA kit with 200 ng input.

In more detail, for each library preparation method, we computed the number of differentially expressed genes at each input amount, using the maximum input for each kit as a reference. This analysis indicates how bioinformatic analyses that rely on differentially expressed genes will be impacted by input mRNA amounts and library preparation methods. We found that Illumina TruSeq and Swift Rapid RNA kits were sensitive to changes in input, with respectively 1,011 and 555 genes differentially expressed between 50 ng and the maximum input at a 0.05 level of significance after adjustment for multiple gene testing (Fig. 6). Under the same conditions, the Swift RNA kit produced one differentially expressed gene.

**Figure 7:**
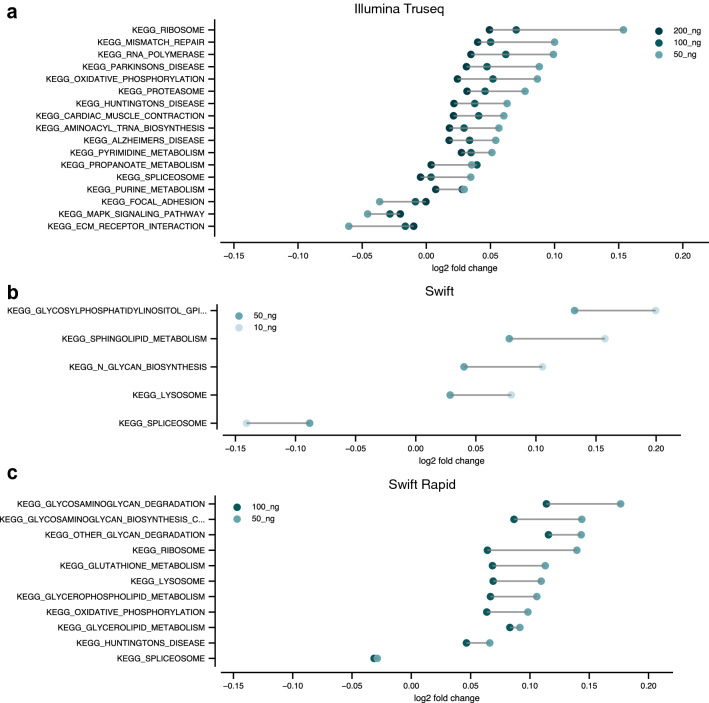
Comparison of differential pathway expression results. Average log_2_ fold change of genes in differentially expressed KEGG pathways at the *p* = .01 significance level (at any input amount) depends on library preparation method and input. Differential expression computed with respect to reference samples prepared with (**a**) the Illumina TruSeq kit with 500 ng input, (**b**) the Swift RNA kit with 100 ng input, and (**c**) the Swift Rapid RNA kit with 200 ng input.

We next wondered if these differentially expressed genes, which plausibly result from technical considerations independent of experimental design, would affect the interpretation of bioinformatic analyses. In particular, we asked if these genes we identified were spread uniformly across the genome or concentrated in particular pathways, which would increase the chance of spurious conclusions. To answer this question, we used the database of human pathways curated by the Kyoto Encyclopedia of Genes and Genome (KEGG)^17^. Using the method described in Leeman D. et al. (2018)^18^, we computed the average fold change of the genes in each of 186 KEGG pathways for each of the input amounts in each library prep method (Fig. 7).

We found all methods performed well. For instance, at a significance level of 0.01 (adjusted for multiple pathway testing), the Illumina TruSeq kit produced 17 differentially expressed pathways, with a maximum log_2_ fold change of approximately 0.15, corresponding to a change of + 11% for the KEGG Ribosome pathway. The Swift RNA and Swift Rapid RNA kits produced similar results, but with fewer differentially expressed pathways (5 and 11, respectively). Interestingly, both the KEGG lysosomal and spliceosomal pathways were significantly perturbed in the Swift kits, while many pathways commonly studied in neuroscience (oxidative phosphorylation, Parkinson's, Huntington's, Alzheimer's) were similarly perturbed in the Illumina TruSeq data. These results suggest that domain specific biases may exist for each library preparation method, and accurate measurement of neuroscience pathways may depend on input RNA amount. However, such biases can be avoided by increasing the amount of input mRNA or, when limited by input material, focusing attention on only those pathways with large fold change (absolute log_2_ fold change > 0.15).

## Discussion

RNA sequencing is a powerful technology commonly used in fundamental biological research as well as clinical applications. High throughput RNA-seq experiments offer a unique opportunity to understand how diverse chemical and genetic perturbations affect the transcriptome. One limitation in performing these experiments is the preparation of cDNA libraries, which remains expensive and labor-intensive and often requires substantial amounts of RNA.

Several low input RNA kits have been compared to the TruSeq mRNA kits, considered to be the gold standard for RNA-seq studies. Systematic analysis of TruSeq SMARTer and SMARTer Ultra-Low RNA-seq kits^2^ show that the SMARTer kit, used in combination with ribodepletion kits, had decreased performance for the inputs of 100 and 10 ng on multiple metrics as compared to the TruSeq kits. Furthermore, when associated with the RiboZero step for ribodepletion, the method is rendered incompatible for automation. In contrast, the SMARTer Ultra-Low kit performed relatively well for ultra-low inputs < 1 ng and is automation-compatible; however, it is associated with higher per sample cost (currently about $100 per sample). Other RNA-seq library preparation methods evaluated for strand specificity and lower input compared the TruSeq kit include the Takara Bio SMART-Seq v4 Ultra Low Input RNA kit (V4) which sacrifices strand specificity, and the SMARTer Stranded Total RNA-Seq Kit v2—Pico Input (Pico) kit^3^. Although the Pico kit retains strand specificity and has good concordance with the TruSeq kit, libraries generated using the Pico kit showed 55% fewer differentially expressed genes^3^. Comparative analysis of the earlier-generation Ovation RNA-seq system with the Illumina TruSeq kits revealed that the kit performed well with almost equal gene representation for low inputs ranging from 500 pg–10 ng total RNA, however, these libraries exhibited a platform-specific 5’ or 3’ bias^4^. While the newer generation NuGen Ovation low input kits have reduced positional bias, they lack strand specificity^1^.

In this work, we have systematically compared three methods—Swift RNA, Swift Rapid RNA and Illumina TruSeq stranded mRNA kits—for developing libraries, focusing on the ease of the workflow, its promise for automation, and the accuracy of resulting bioinformatic analyses. The Swift RNA library kit accommodates a broader input range and offers consistent and reliable results across diverse samples. This is achieved through the use of the patented Adaptase technology which allows sequential adapter ligation and helps avoid adapter titration across inputs. This maintains uniform adapter concentrations at all inputs. Additionally, Adaptase performs simultaneous tailing and ligation of NGS adapters to ssDNA. Adaptase also allows for DNA inputs as low as a single cell and its high efficiency template-independent chemistry provides comprehensive coverage with minimal base composition bias. This technology supports a broad input range down to 10 ng total RNA. Consistent performance from damaged samples such as FFPE RNA has also been reported.

The differences in workflow time across the Illumina and Swift kits are attributed to different kit chemistries. In the Illumina TruSeq Stranded mRNA kit, strand specificity is enforced by replacing dTTP with dUTP, followed by second strand cDNA synthesis using DNA Polymerase I and RNase H. This method works by employing Uracil-DNA-Glycosylase to excise the newly incorporated uracil base on the second strand, thus blocking this strand from being used as a template in PCR. On the other hand, the faster workflow for the Swift kits is facilitated by the proprietary Adaptase technology which supports library preparation from first strand cDNA, thereby eliminating the need for second strand synthesis. Furthermore, the Swift RNA workflow is also compatible with low quality, low RIN FFPE samples. While the Swift Rapid RNA method is the fastest workflow, it is best suited for samples with ample RNA yields.

All methods we examined here produced high quality libraries, as measured by sequencing and alignment metrics. Further, quantified gene counts produced by these methods were in substantial agreement. However, these methods differed in their sensitivity to input RNA amount. Using the manufacturer-recommended RNA input amount as a reference, we assessed the accuracy of differentially expressed genes at 50 ng, a yield reasonably produced for instance by in vitro stem cell models. We found the Swift RNA kit to produce the fewest “false positives”, genes differentially expressed due to technical considerations only, at typical levels of significance used in such analyses. Interestingly, the dependency of differential expression of genes and pathways on input amount differed between the kits we considered. The Swift RNA kit produced the fewest number of differentially expressed KEGG pathways with respect to input amount. Our study suggests that to avoid conclusions based on technical artifacts, researchers should focus on changes in expression of pathways of at least 10%. Furthermore, as KEGG pathways are commonly used in genomics studies as a reference to annotate gene sets, understanding the mechanism by which these biases originate would be a valuable endeavor for future work.

## Methods

SPRIselect beads from Beckman Coulter (B23318) were used for all magnetic bead DNA clean up steps. Universal Human Reference RNA (UHRR) (ThermoFisher, QS0639) was diluted to 100 ng/ul with low TE buffer, aliquoted, and stored at − 80 °C.

### Library preparation

- **Swift RNA library prep. (IDT, xGen Broad-range RNA Library Prep Kit; Cat. No. 10009813, 10010145):** 100, 50, and 10 ng of UHRR was used as direct input for NEBNext Poly(A) mRNA Magnetic Isolation Module (New England Biolabs, Cat. No. E7490). In the final elution step, mRNA was eluted off the beads into the IDT xGen Broad-range RNA Library Kit Fragmentation Buffer. Libraries were prepared and indexed (IDT xGen Unique Dual Indexing Primer Plate 2-8; Cat. No. 10009816) using Swift recommended protocol. cDNA libraries were diluted 1:5 in low TE buffer and analyzed on Agilent 4200 TapeStation using D5000 HS DNA screentape. Libraries that gave yields < 4 nM were amplified using the IDT Library Amplification Primer Mix (Cat. No. 10009867).
- **Swift Rapid RNA library prep. (IDT, xGen RNA Library Prep Kit; Cat. No. 10009814, 10010146):** 200, 100 and 50 ng of UHRR was used as direct input for NEBNext Poly(A) mRNA Magnetic Isolation Module (New England Biolabs, Cat. No. E7490). In the final elution step, mRNA was eluted off the beads into the IDT xGen RNA Library Kit Fragmentation Buffer. Libraries were prepared and indexed (IDT xGen Unique Dual Indexing Primer Plate 2-8; Cat. No. 10009816) using Swift recommended protocol. cDNA libraries were diluted 1:1 in low TE buffer and analyzed on Agilent 4200 TapeStation using D5000 DNA screentape.
- **Illumina TruSeq stranded mRNA library Prep. (Illumina, 20020594, 20020595):** 500, 200, 100 and 50 ng of UHRR was used as direct input for the Illumina TruSeq Stranded mRNA (20020594). Libraries were prepared and indexed (IDT for Illumina-TruSeq RNA UD Indexes, 20022371) as outlined in the TruSeq Stranded mRNA Reference Guide.

### Library QC and quantification

Final libraries were diluted in low TE buffer and analyzed on the Agilent 4200 TapeStation using D5000 or D1000 DNA screentape. Library quantification was performed using KAPA qPCR. Six serially pre-diluted DNA standards and 1:10,000 or 1:20,000 diluted NGS libraries were amplified using platform-specific qPCR primers that target adapter sequences as per manufacturer’s instructions using *N* = *3* technical replicates per sample. The standard curve generated using the six pre-diluted DNA standards is used to convert the average Cq value for diluted libraries to concentration, from which the working concentration of each library is calculated.

### Sequencing

Prepared libraries were pooled at 10 nM concentration each and sequenced using the NovaSeq SP sequencer (200 cycles, with XP workflow), with a 2 × 100 bp paired-end read length and an average read depth of 20 million read pairs per library. Sequencing was performed at Seqmatic LLC (Fremont, California, USA).

### Alignment and quantification

10 million read pairs for each library were randomly sampled without replacement to account for differences in sequencing depth using the open-source software seqtk^22^. Subsampled reads were aligned with STAR version 2.7.2 to the reference genome GRCh38 with Ensembl gene annotations^13,19^ and the flags --outSAMtype BAM SortedByCoordinate --quantMode GeneCounts. We required genes to have a minimum 3 counts per million in at least 20% of samples. Genes not satisfying this requirement were removed. After filtering, approximately 12,000 genes remained. To compare absolute gene expression, reads were normalized via the regularized log transform^20^.

### Differential expression of genes

Each of these sample groups was compared to the reference as specified in the main text using the linear model *Counts* ~ *Treatment group.* Log_2_ fold changes and significance were computed with the R software package DESeq2^20^. *p* values were adjusted for multiple hypothesis testing by the Benjamini–Hochberg method^21^.

### Differential expression of pathways

To test for pathway enrichment among differentially expressed genes, we collected a set of gene pathways curated by KEGG and available from MSigDB^17^ (file name: c2.cp.kegg.v7.1.entrez.gmt). The statistical test for enrichment of these pathways among differentially expressed genes is due to Leeman et al.^18^. In this method, the test statistic for a gene set is the average *t*-statistic from DESeq2 of all genes in the set, and a permutation test is used to approximate the distribution of this test statistic. The null hypothesis for this test is that the genes in a given gene set show the same pattern of association with the phenotype compared with the rest of the genes. *p* values were adjusted for multiple hypothesis testing by the Benjamini–Hochberg method^21^.

## Supplementary Information

Supplementary Information.

## Data Availability

Sequencing data is available at the NIH GEO website (accession number GSE167300). Code for the analysis is available at https://github.com/rajbhatnagar/LibraryPrep2021.
