# Supplementary material for: Systematic comparative analysis of strand-specific RNA-seq library preparation methods for low input samples
Source: Sci Rep. 2022 Feb 2;12:1789. doi: 10.1038/s41598-021-04583-z (PMC8810888; doi:10.1038/s41598-021-04583-z)
Supplement: Supplementary file 1 — Supplementary Information. [file 41598_2021_4583_MOESM1_ESM.pdf]

Supplemental Figure 1

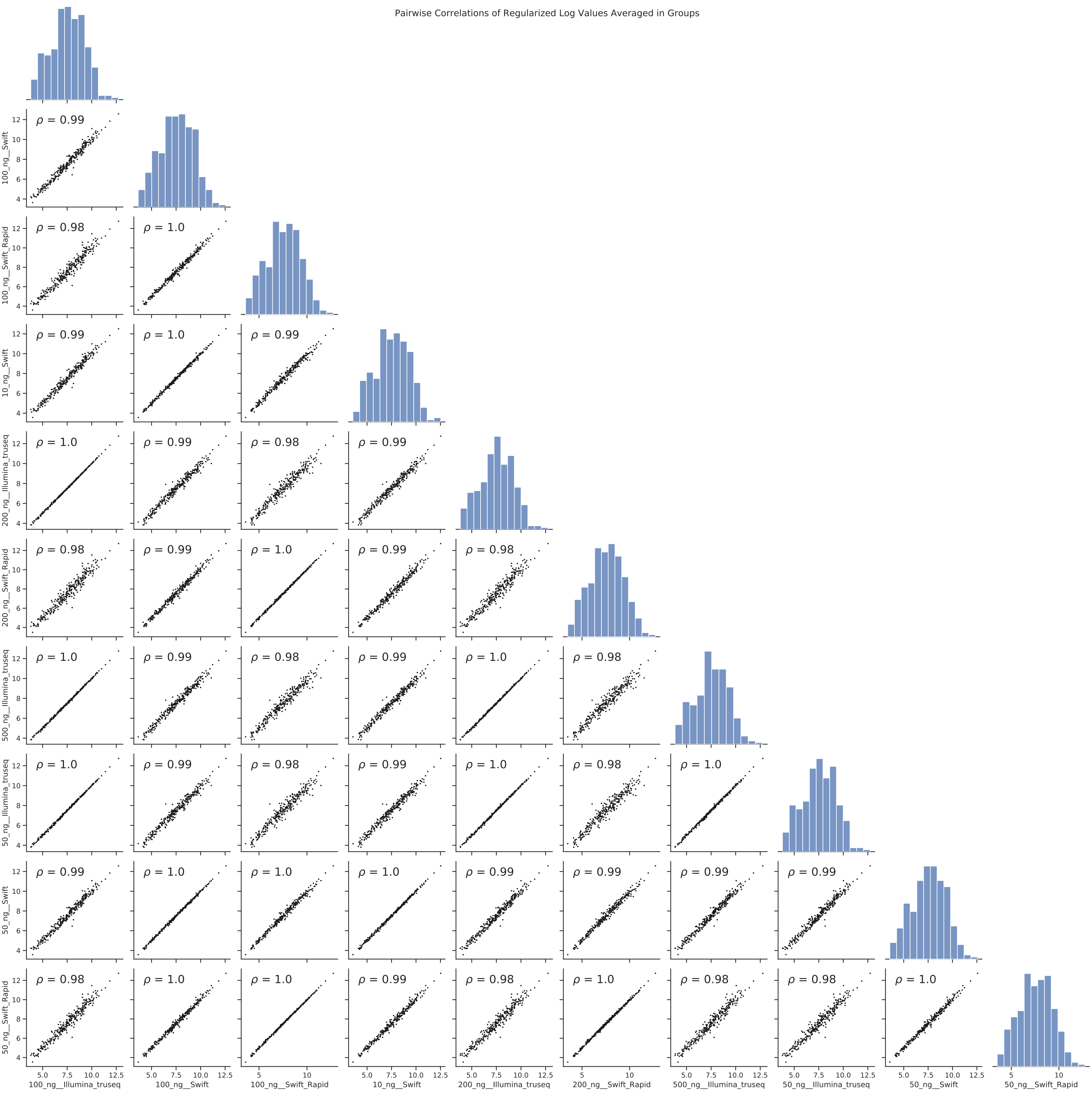

Supplemental Figure 1: Normalized read counts from identical samples prepared using three commercially available methods (Illumina TruSeq, Swift RNA, and Swift Rapid RNA) at multiple RNA amounts are highly correlated (c.f. Figure 3b)

Supplemental Figure 2

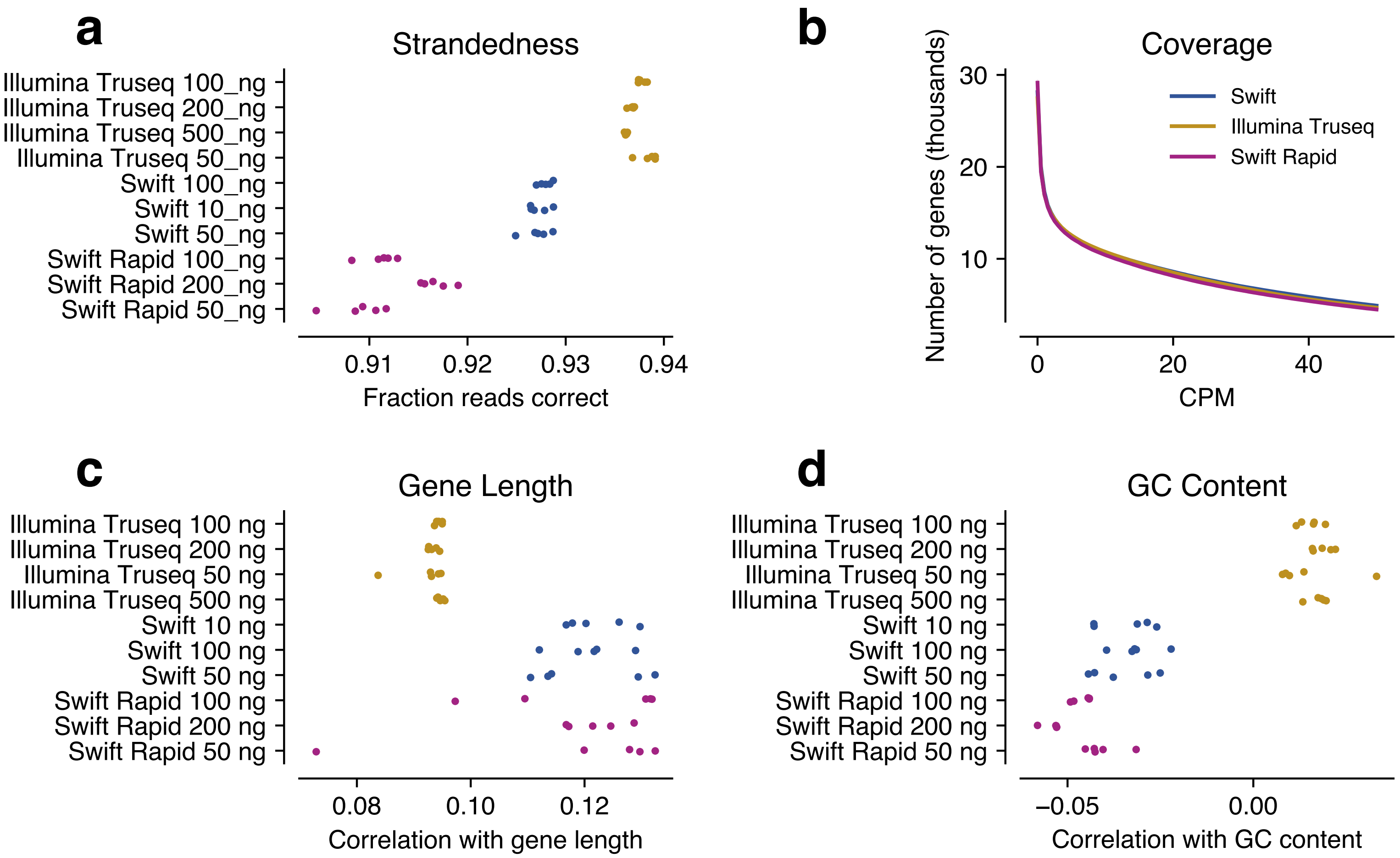

Supplemental Figure 2: (a) Fraction of reads that map to the correct strand exceeds 90% for all samples. (b) Library complexity, as measured by the number of genes detected (y-axis) that exceed a threshold (x-axis) less than 50 counts per million, is comparable across all three methods. (c) Correlation of gene expression values with gene length for each sample. (d) Correlation of gene expression values with gene GC content for each sample.

Supplemental Figure 3

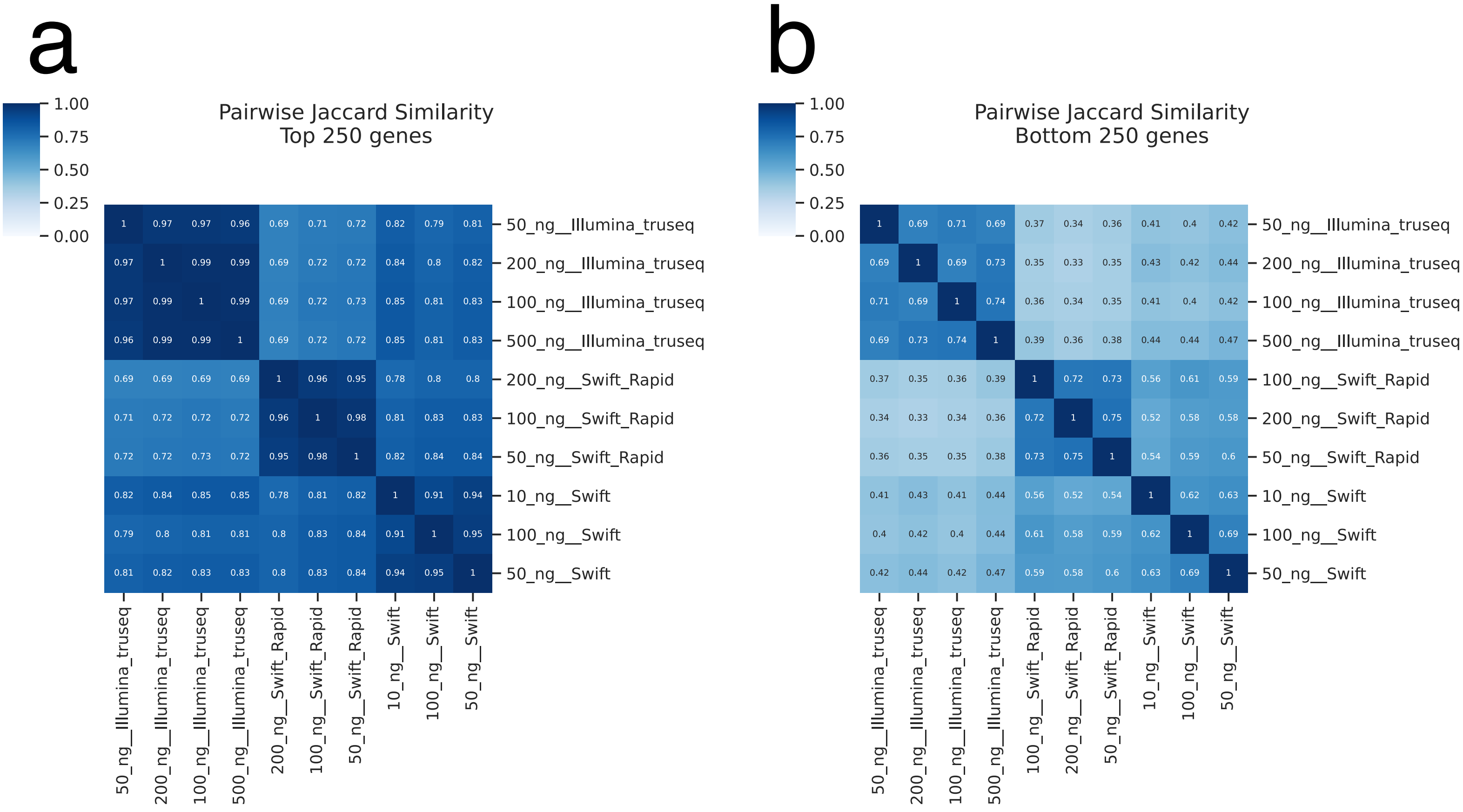

Supplemental Figure 3: Jaccard similarity coefficient between the (a) the 250 highest expressed genes for each method and input amount and (b) the 250 lowest expressed genes for each method and input amount.

Supplemental Figure 4

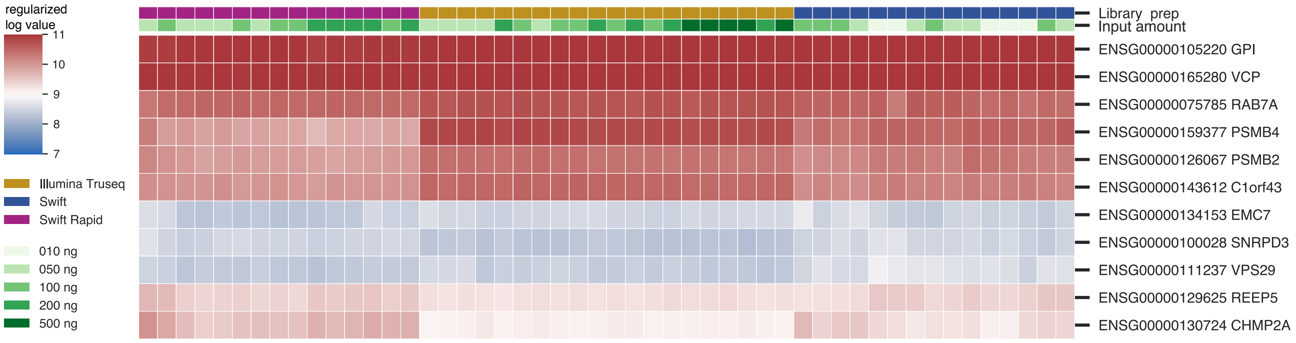

Supplemental Figure 4: Absolute expression of housekeeping genes with different library preparation kits and across different input amounts. Genes and methods are hierarchically clustered using the Euclidean metric.

Supplemental Figure 5

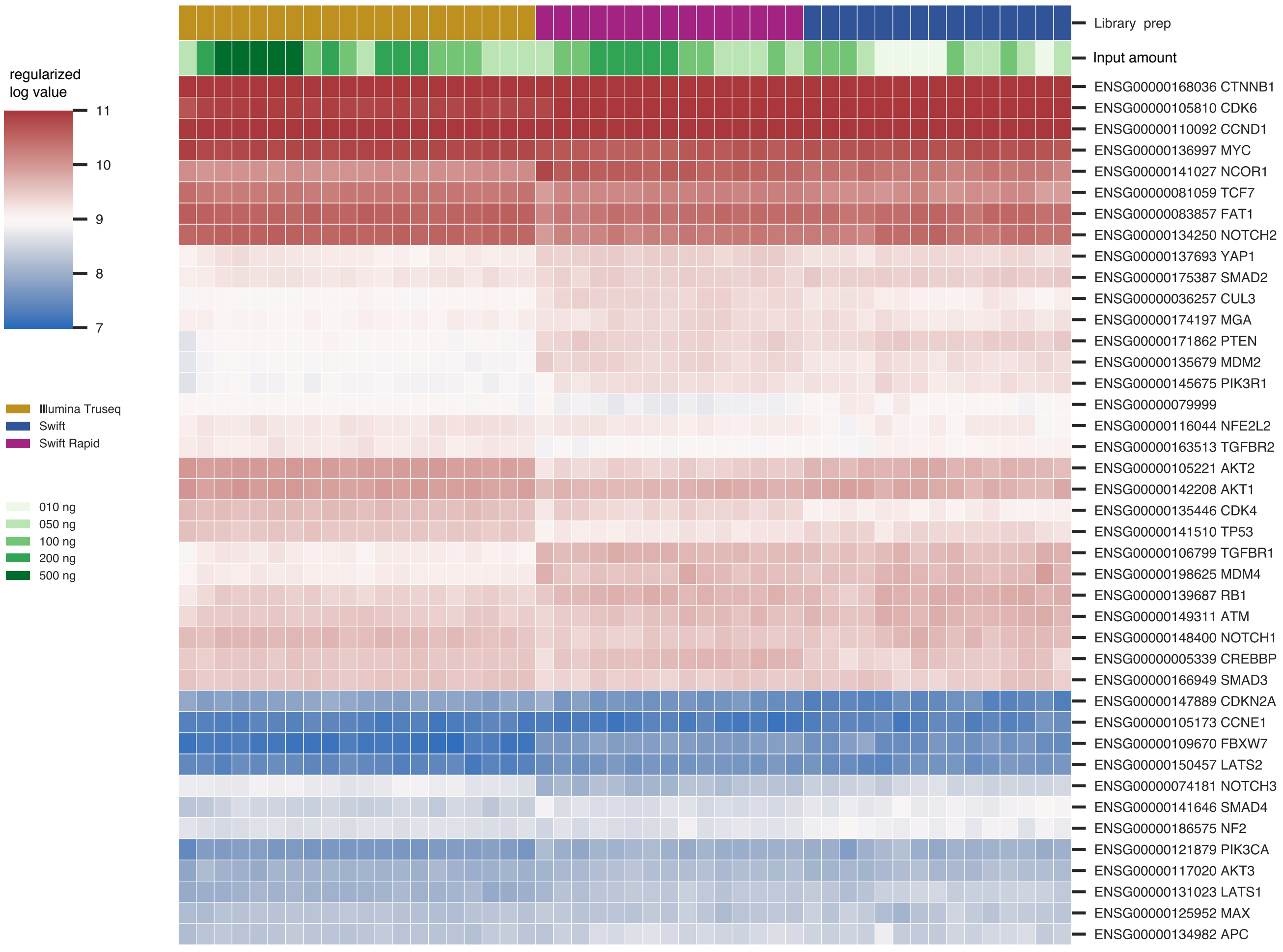

Supplemental Figure 5: Absolute expression of oncogenic signaling pathway genes with different library preparation kits and across different input amounts. Genes and methods are hierarchically clustered using the Euclidean metric.
